# Multiple Regulatory Mechanisms to Inhibit Untimely Initiation of DNA Replication Are Important for Stable Genome Maintenance

**DOI:** 10.1371/journal.pgen.1002136

**Published:** 2011-06-16

**Authors:** Seiji Tanaka, Hiroyuki Araki

**Affiliations:** 1 Division of Microbial Genetics, National Institute of Genetics, Mishima, Japan; 2 Department of Genetics, School of Life Science, The Graduate University for Advanced Studies (SOKENDAI), Mishima, Japan; National Cancer Institute, United States of America

## Abstract

Genomic instability is a hallmark of human cancer cells. To prevent genomic instability, chromosomal DNA is faithfully duplicated in every cell division cycle, and eukaryotic cells have complex regulatory mechanisms to achieve this goal. Here, we show that untimely activation of replication origins during the G1 phase is genotoxic and induces genomic instability in the budding yeast *Saccharomyces cerevisiae*. Our data indicate that cells preserve a low level of the initiation factor Sld2 to prevent untimely initiation during the normal cell cycle in addition to controlling the phosphorylation of Sld2 and Sld3 by cyclin-dependent kinase. Although untimely activation of origin is inhibited on multiple levels, we show that deregulation of a single pathway can cause genomic instability, such as gross chromosome rearrangements (GCRs). Furthermore, simultaneous deregulation of multiple pathways causes an even more severe phenotype. These findings highlight the importance of having multiple inhibitory mechanisms to prevent the untimely initiation of chromosome replication to preserve stable genome maintenance over generations in eukaryotes.

## Author Summary

Chromosomal DNA replication occurs as a two-step reaction in eukaryotes. In the first reaction, called licensing, the replicative helicase is loaded onto replication origin in an inactive form during the G1 phase of the cell cycle. In the second reaction, called initiation, the replicative helicase is activated, and replication forks are established. Because of this two-step mechanism, licensing and initiation must occur at different times in the cell cycle. Failure of this two-step regulation will cause heterogeneous re-replication of chromosomal DNA, and genome integrity will be lost. Although previous works have established that multiple regulatory pathways regulate licensing, much less is known about how untimely (premature) initiation is prevented during the G1 phase. In this paper, we show that untimely activation of replication origins during the G1 phase is inhibited on multiple levels. Notably, deregulation of a single pathway can cause genomic instability; simultaneous deregulation of multiple pathways causes a more severe phenotype, such as aneuploidy. Therefore, these findings not only indicate the importance of having multiple inhibitory mechanisms to prevent untimely initiation of chromosome replication but also should help us understand how replication might be deregulated in human cancer cells, in which the genome is frequently destabilized.

## Introduction

When eukaryotic cells proliferate, their chromosomes must be precisely duplicated and segregated to daughter cells to maintain genome stability over generations. Failure of these processes is directly connected to lethality and severe disease, such as cancer. Genome instability is a hallmark of human cancer cells. To duplicate chromosomal DNA precisely, DNA replication must be restricted to occur exactly once per cell cycle. Chromosomal DNA replication in eukaryotes initiates from multiple specific regions of chromosomal DNA, called origins of replication. Therefore, it is important to regulate the activation of replication origins to only once per cell cycle because multiple rounds of origin activation per cell cycle will cause over-replication. Such re-replication might cause copy number heterogeneity throughout the genome, and genome integrity will be lost.

In eukaryotes, replication origin activation occurs as a conserved two-step reaction (for reviews, see [1]–[3]). In the first reaction, known as licensing, a specific protein-origin DNA complex, called the pre-replicative complex (pre-RC), is assembled at origins during the G1 phase of the cell cycle by the loading of an inactive form of the Mcm2-7 helicase complex. In the second reaction, called initiation, the pre-RC is activated, and bidirectional replication forks are established for DNA synthesis. Because of this two-step DNA replication initiation mechanism, these two reactions must occur in separate cell cycle phases. Therefore, initiation does not occur when cells assemble pre-RCs in G1 phase, and pre-RC assembly is inhibited when initiation can occur from S to M phase. Thus, DNA replication is limited to once per cell cycle.

Eukaryotes have multiple mechanisms to prevent pre-RC re-assembly at activated origins, [4]. For example, in the budding yeast *Saccharomyces cerevisiae*, all components of the pre-RC, including ORC, Cdc6, Cdt1, and Mcm2-7, are inhibited by the master cell cycle regulator, cyclin-dependent kinase (CDK). ORC is inhibited by CDK through phosphorylation of Orc2 and Orc6, and the S-phase cyclin Clb5 binds directly to an RXL motif in Orc6 [5], [6]. Cdc6 is also inhibited by CDK in three ways: transcription, proteolysis and direct association with mitotic CDK [7]–[9]. Finally, nuclear accumulation of Mcm2-7 and Cdt1 is inhibited by CDK activity [10]–[12]. Each of inhibitory reaction contributes to prevent pre-RC formation. Because of these multiple down-regulatory mechanisms, the deregulation of any one mechanism does not cause a severe phenotype. However, the simultaneous deregulation of more than one mechanism causes a more severe phenotype. Finally, the simultaneous deregulation of all of the mechanisms causes robust DNA re-replication [5], [9]. Because any one mechanism is insufficient to inhibit pre-RC formation completely, it is important to have multiple mechanisms to strictly enforce once per cell cycle DNA replication. Indeed, multiple inhibitory pathways for the formation of the pre-RC are common in model eukaryotes, although the specific mechanisms are different between organisms [4].

Untimely activation of the pre-RC during G1 phase must also be prevented because origin firing in G1 results in the reformation of pre-RC at replicated origin DNA, leading to multiple rounds of replication of the region [13], [14]. CDK and DDK (Dbf4-dependent kinase, which consists of Cdc7 and Dbf4) are conserved protein kinases and are required for activation of the pre-RC in eukaryotes [3]. Until recently, budding yeast was the only organism in which the essential targets of CDK in initiation had been identified. In this organism, S phase-specific CDKs (S-CDKs: Clb5- and Clb6-Cdc28) phosphorylate two essential replication proteins, Sld2 and Sld3, to promote DNA replication [13]–[15]. Phosphorylation of Sld2 and Sld3 enhances the interaction between Sld2 or Sld3 and a third protein Dpb11, respectively [13]–[16]. These interactions are not only essential for initiation but are also sufficient to bypass the requirement for CDK in the initiation of DNA replication. Combinations of mutations that can bypass the CDK phosphorylation of Sld2 and Sld3 allow cells to promote DNA replication [13], [14]. For example, CDK phosphorylation of Sld2 and Sld3 can be bypassed by phosphomimetic form of Sld2 and the Cdc45^Jet1-1^, respectively [13]. When phosphomimetic Sld2 (Sld2-11D) is induced from a galactose-inducible promoter (*GALp*) in the *CDC45^JET1-1^* background, DNA replication occurs even in G1-arrested CDK inactive cells. Under this condition, DNA re-replication occurs as expected, indicating that repeated formation and activation of the pre-RC is occurring [13].

This “CDK-bypass” DNA replication, surprisingly, requires neither bypass of DDK nor artificial expression of Dbf4 [13]; however, it is inhibited by inactivation of DDK. Although DDK's regulatory subunit, Dbf4, is degraded via anaphase promoting complex/cyclosome (APC/c) in G1 phase [17]–[19], these observations suggest that G1 cells have residual DDK activity and that activity is sufficient to induce DNA replication [13]. This further suggests that CDK activity is crucial for the initiation of DNA replication during G1 phase. We thus examined a CDK-bypass strain to elucidate the consequences of untimely initiation and how it is prevented in wild-type yeast cells. Our results show that untimely initiation in G1 causes a severe loss of viability and the genomes of surviving cells are very frequently destabilized. To prevent untimely initiation and to maintain genome stability, multiple mechanisms are employed.

## Results

### Cells Are Sensitive to Untimely DNA Replication in G1

Untimely activation of the pre-RC in G1 phase causes multiple rounds of origin activation. To understand the effect of untimely DNA replication in G1 on cell viability, we induced untimely DNA replication in G1 through the high-level expression of phosphomimetic Sld2 in *CDC45^JET1-1^* cells and monitored cell viability. Cells were arrested and kept in G1 phase with alpha factor, and then, various Sld2 derivatives were expressed. Only when phosphomimetic Sld2s (Sld2-11D: all potential 11 CDK phosphorylation sites are substituted by aspartic acid and Sld2-T84D: only essential threonine 84 is substituted by aspartic acid) were expressed did DNA replication occur as previously shown (Figure S1A). Because cells were kept in alpha factor-containing medium, S-CDK was not activated, and Orc6 protein, a phosphorylation target of S-CDK [5], was maintained in a hypophosphorylated fast-migrating form during the experiment (Figure S1C). When DNA replication occurred in phosphomimetic Sld2-expressing cells, their viability was rapidly diminished (Figure S1B, S1C). For example, one hour after Sld2-11D and Sld2-T84D induction, the increased DNA content estimated from flow cytometry was only approximately 10% and 1.5% (see Materials and Methods for details), but 93% and 85% of cells lost viability, respectively (Figure S1A, S1B). When origin firing is induced by phosphomimetic Sld2 and Jet1-1, pre-RCs re-assemble again at origins because in alpha factor-arrested cells, low CDK activity allows pre-RC formation in budding yeast. Therefore, untimely replicated portions of chromosomes will be replicated repeatedly in the same G1 phase or in the following S phase. It has been shown that multiple rounds of DNA replication (re-replication) occur during CDK-bypass replication [13]. Therefore, our results imply that cell viability is very sensitive to re-replication.

To further confirm that untimely replication causes a loss in viability, we utilized a *cdc7-4* temperature-sensitive allele of *CDC7* that is defective in the catalytic subunit of DDK. Previously, it has been shown that DDK activity is required for CDK-bypass DNA replication in *CDC45^JET1-1^ GALp-sld2-11D* cells [13]. At the restrictive temperature, *cdc7-4* inhibits CDK-bypass DNA replication [13]. *cdc7-4 CDC45^JET1-1^ GALp-sld2-11D* cells were first arrested in G1 with alpha factor and then kept in alpha factor-containing medium until the end of the experiment to maintain low CDK activity. After G1 synchronization, the temperature was shifted to the restrictive temperature (37°C) for *cdc7-4*, and then Sld2-11D was expressed by the addition of galactose. High-temperature incubation not only prevented untimely DNA replication but also inhibited the loss of viability (Figure S2). In contrast, at 25°C, DNA replication occurred, and in addition, viability was lost when galactose was added (Figure S2). Therefore, we conclude that the loss of viability observed here is caused by untimely DNA replication during G1 phase rather than the high level of phosphomimetic Sld2 itself.

CDK-bypass DNA replication does not require artificial expression of Dbf4 because G1 cells have residual DDK activity as described above [13]. Moreover, ectopic expression of Dbf4 enhances the extent of DNA replication [13], which further predicts that the expression of Dbf4 in G1-arrested cells leads to more re-replication and a greater loss in viability. To test this possibility, we simultaneously induced Dbf4 from a galactose-inducible promoter with Sld2-11D in *CDC45^JET1-1^ GALp-sld2-11D* cells (Figure S3). Although the effect was not strong, Dbf4 expression resulted in more DNA replication and loss of viability (Figure S3). These results further indicate that the loss in viability observed here is a direct consequence of re-replication.

### Untimely DNA Replication in G1 Induces Genomic Instability

**Figure 1:**
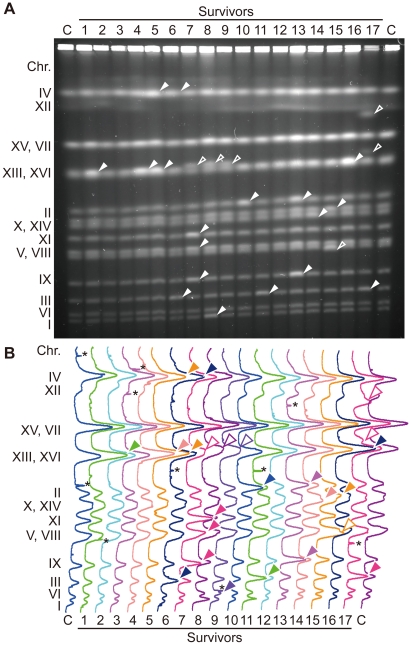
Survivors of the untimely initiation of DNA replication experiments have aberrant chromosomes. A, Chromosomal DNA from YST563 (*JET1-1 GALp-sld2-11D*) survivors from Figure S1 was analyzed using pulsed-field gel electrophoresis. Surviving colonies (survivors 1–17) were recovered on YPAD plates after galactose incubation. Abnormal chromosome bands are indicated with arrowheads, chromosomes with increased band intensity are indicated with filled arrowheads, and chromosomes with different lengths are indicated with open arrowheads. Chr XII bands are not marked because they are unstable even in wild-type cells. B, Quantified profiles of band intensities of A are shown. Abnormal chromosome bands are indicated with arrowheads. *: non-chromosomal signal originated from contaminated dusts.

Although untimely initiation induced in G1-arrested cells killed most of *CDC45^JET1-1^ GALp-sld2-11D* cells (Figure S1B), small numbers of surviving cells were recovered on glucose-containing plates. Similar to the original cells, these surviving cells were unable to grow on the galactose-containing plate. To examine the effect of DNA re-replication on genome stability, we analyzed the chromosomes of survivors by pulsed-field gel electrophoresis (Figure 1A). In the wild-type control cells, of 10 clones examined, no survivors showed gross chromosome abnormalities except for fluctuation in the length of chromosome XII (Figure S4A). Chromosome XII harbors the rDNA repeats, and the rDNA copy number fluctuates naturally [20], [21]. Therefore, we omitted chromosome XII from the analysis. In contrast to wild-type cells, *CDC45^JET1-1^ GALp-sld2-11D* survivors had an abnormal chromosome composition. For example, in survivors 6, 11 and 17 (Figure 1, lanes 6, 11 and 17), the band intensity ratio of chromosome III was almost two times higher than that of the control (lane C at both ends), indicating a duplicated chromosome III. In addition, much aneuploidy was observed (Figure 1, filled arrowheads). Moreover, some other survivors appeared to have chromosomes with an atypical length (Figure 1, lanes 7, 8, 9, 15 and 17, open arrowheads). Of 17 clones examined, 14 showed chromosome abnormalities (Figure 1). These data show that untimely DNA replication induces abnormal chromosome composition. *CDC45^JET1-1^ GALp-sld2-11D* cells maintained high viability when Sld2-11D was not induced (Figure S1B). However, four of 12 clones obtained from this condition showed an abnormal chromosome composition (Figure S4B, data not shown). This result suggests that chromosome composition is frequently altered in *CDC45^JET1-1^ GALp-sld2-11D* cells, even when the cells retain high viability.

### The G1/S Phase Level of Sld2-11D Can Induce Untimely DNA Replication

**Figure 2:**
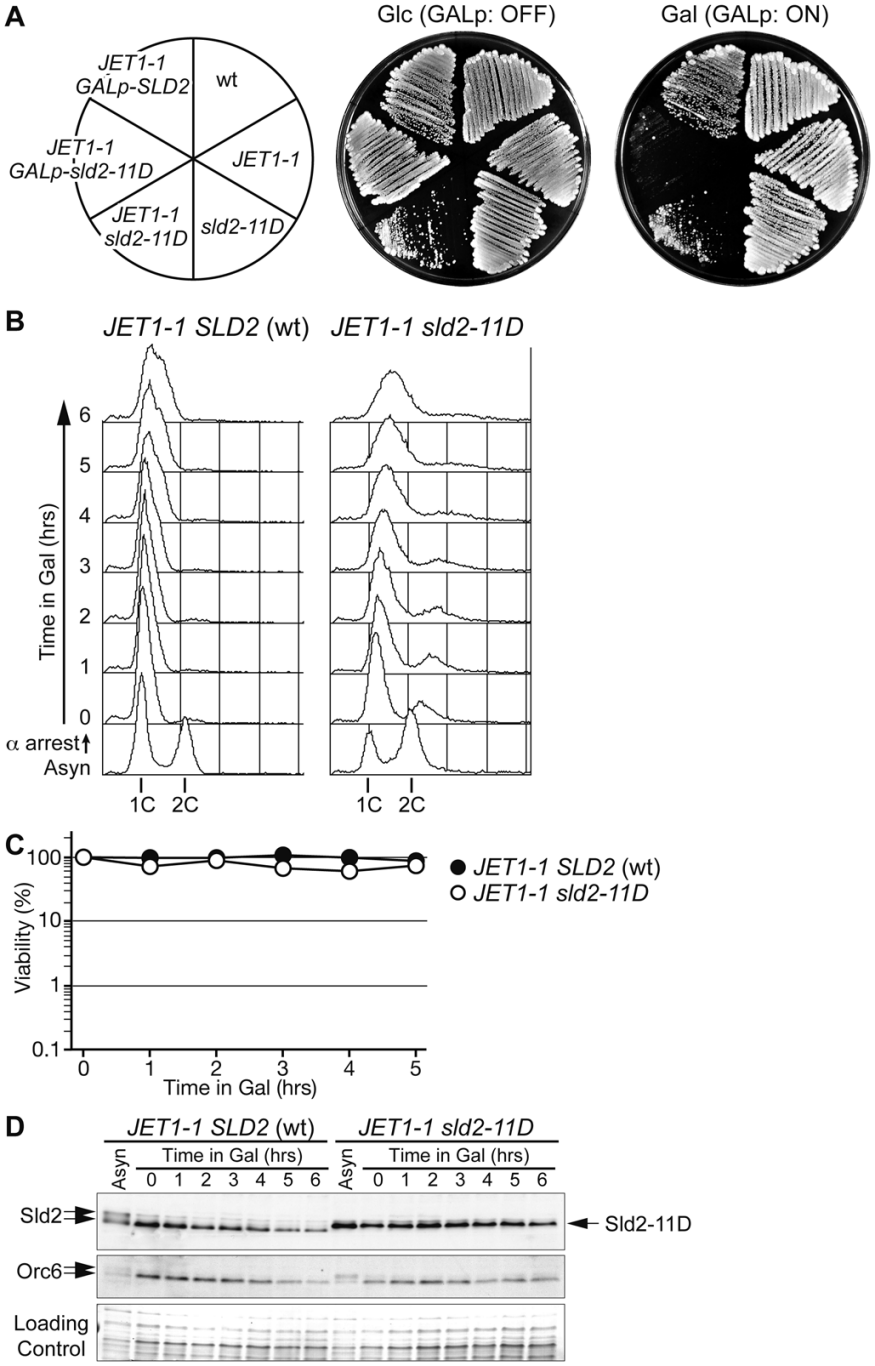
Endogenous levels of Sld2-11D do not promote untimely DNA replication efficiently. A, W303-1a *Δbar1* (wt), YST556 (*JET1-1*), YST831 (*sld2-11D*), YST816 (*JET1-1 sld2-11D*), YST562 (*JET1-1 GALp-sld2-11D*) and YST560 (*JET1-1 GALp-SLD2*) were grown on YPAD (Glc) or YPAGal (Gal). B, YST827 (*JET1-1 SLD2* (wt)) and YST829 (*JET1-1 sld2-11D*) cells were grown in YPARaffinose medium (Asyn), arrested in G1 phase with alpha factor, galactose was added and samples were taken at the indicated times. The DNA contents of the samples were analyzed by flow cytometry. C, Small aliquots of the same samples from B were spread onto YPAD plates, and the viability was calculated from the number of colonies that appeared after incubation. D, Whole cell extracts were prepared from the same samples from B and were analyzed by western blotting. Sld2 and Orc6 proteins were detected with anti-Sld2 and anti-Orc6 antibodies, respectively. The loading control shows the corresponding region of the Ponceau-S-stained membrane.

As shown above, the expression of phosphomimetic Sld2, Sld2-T84D or Sld2-11D is a key to the induction of untimely DNA replication in the *CDC45^JET1-1^* background. To further understand the conditions for untimely DNA replication, we next tried to replace the genomic copy of *SLD2* with *sld2-11D* in the *CDC45^JET1-1^* background. We first expected that the *sld2-11D CDC45^JET1-1^* strain could not be isolated because of the re-replication phenotype. Surprisingly, we were able to isolate the *sld2-11D CDC45^JET1-1^* strain, although the cells grew very slowly (Figure 2A). Moreover, the cells arrested in G1 did not replicate DNA (Figure 2B) or lose viability (Figure 2C), although a significant amount of Sld2-11D protein was observed (Figure 2D).

**Figure 3:**
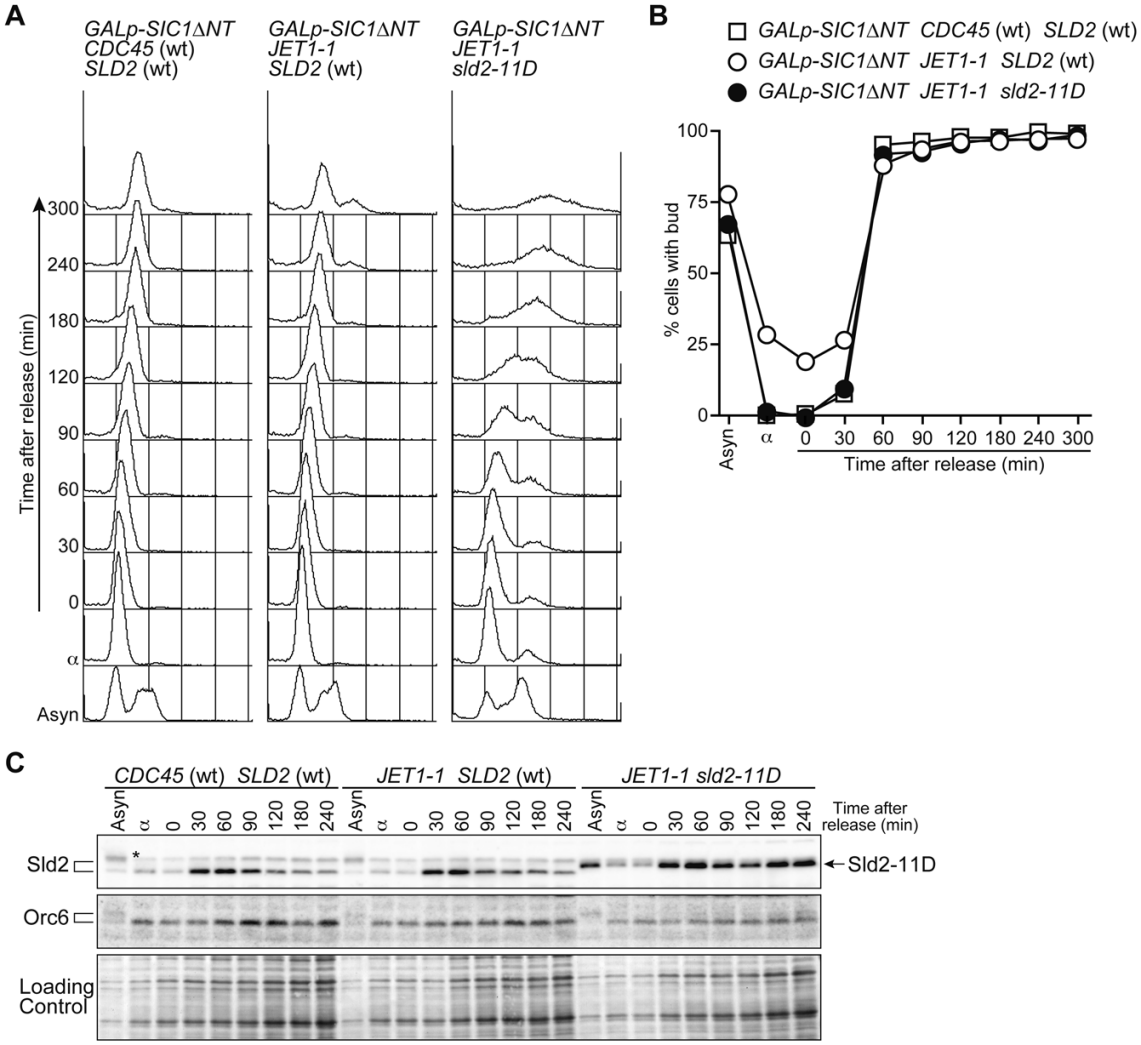
G1/S-level Sld2-11D can promote DNA replication. A, YST1332 (*GALp-SIC1ΔNT CDC45* (wt) *SLD2* (wt)) YST827 (*GALp-SIC1ΔNT JET1-1 SLD2* (wt)) and YST829 (*GALp-SIC1ΔNT JET1-1 sld2-11D*) cells were grown in YPARaffinose medium (Asyn) and arrested in G1 phase with alpha factor (α). Galactose was added, and cells were incubated for 1 hour to express Sic1ΔNT. Cells were then released into fresh YPAGal and collected at the indicated times after release (0–300 min). The samples were analyzed by flow cytometry. B, The proportion of budded cells (YST1332: open boxes; YST827: open circles; YST829: filled circles) at the indicated times are shown. C, Whole cell extract was prepared from the same samples as A and analyzed by western blotting. Sld2 proteins and Orc6 were detected with anti-Sld2 and anti-Orc6 antibodies, respectively. The loading control shows the corresponding region of the Ponceau-S-stained membrane.

The transcript level of *SLD2* fluctuates during the cell cycle and peaks at the G1/S boundary, leading to fluctuation in the Sld2 protein level and its accumulation in S phase [15], [22]. We thus examined whether or not the S-phase level of Sld2 efficiently induces untimely DNA replication. To obtain the S-phase level of Sld2 in the absence of S-CDK activity, we expressed a stable form of Sic1, Sic1ΔNT [23], in G1 phase cells released from alpha factor arrest. Expression of Sic1 inhibits S-CDK activity but not G1-specific CDK (G1-CDK: Cln-CDK in the budding yeast) activity, which induces *SLD2* transcription at the G1/S boundary. As a consequence, *SLD2* is expressed at the normal level seen in S-phase, even in the absence of S-CDK activity. *sld2-11D CDC45^JET1-1^ GALp-SIC1ΔNT* cells were first arrested in G1 with alpha factor, and Sic1ΔNT was then induced before transfer into fresh medium lacking alpha factor. Control cells harboring wild-type *SLD2* arrested at the G1/S boundary because of the high level of Sic1ΔNT, and DNA replication did not occur (Figure 3A). In contrast, DNA replication occurred in cells harboring *sld2-11D* (Figure 3A). The percentage of budded cells, which is indicative of G1-CDK activity, increased 30 to 60 minutes after release from the G1 block in all strains (Figure 3B). Reflecting this result, the Sld2-protein level increased in all strains (Figure 3C), whereas DNA replication occurred only in the *sld2-11D* cells (Figure 3A). Therefore, these results suggest that untimely DNA replication requires a higher level of Sld2-11D than is normally seen during early G1 phase.

**Figure 4:**
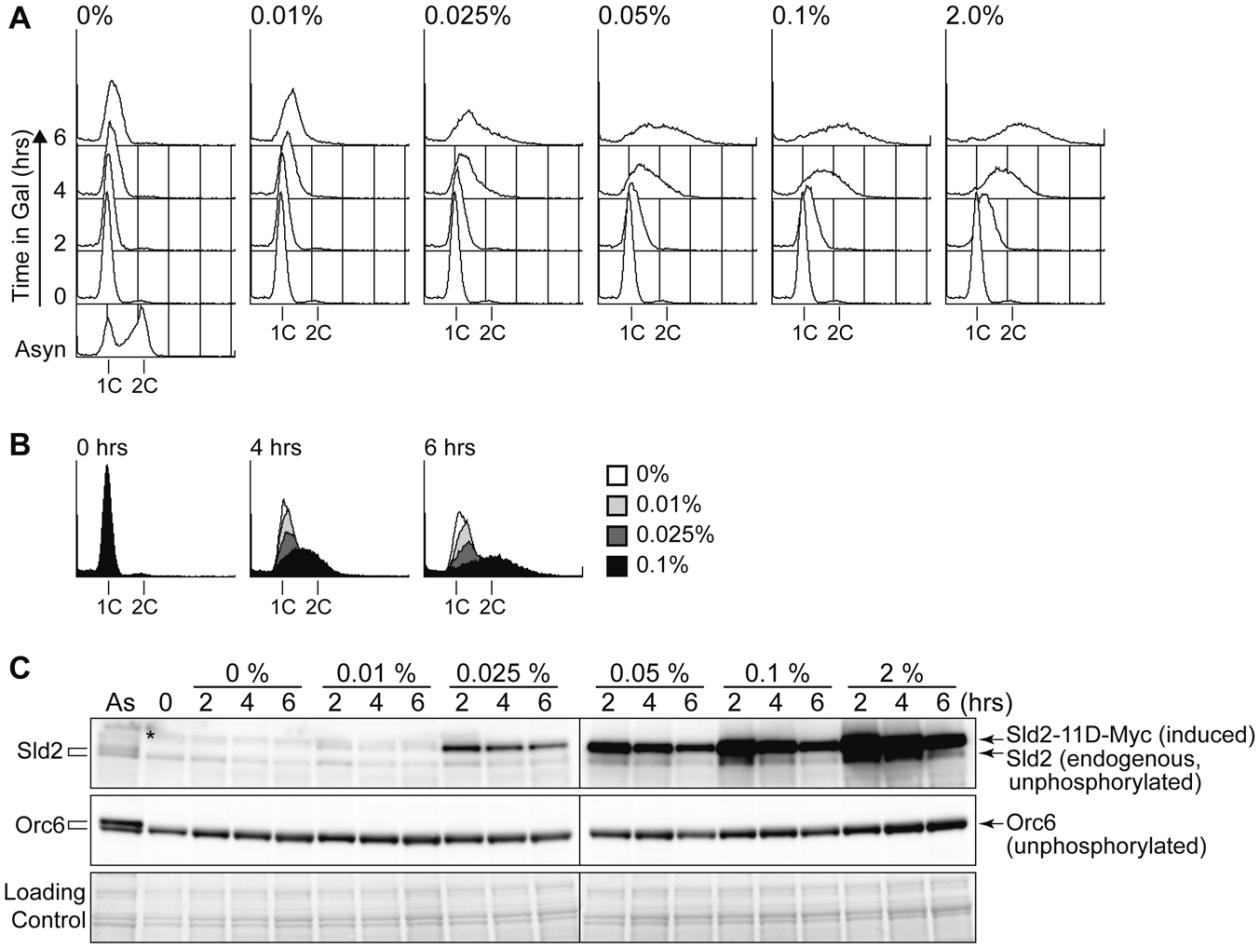
The Sld2-11D protein level is important to promote DNA replication. A, YST1698 (*JET1-1 GALp-sld2-11D ORC6-FLAG*) cells were grown in YPARaffinose medium (Asyn) and arrested in G1 phase with alpha factor, and then the culture was split into six portions. Different amounts of galactose were added to each portion, and samples were taken at the indicated times (0–6 hours after galactose addition). The DNA contents of the samples were analyzed by flow cytometry. B, DNA content at 0, 4 and 6 hours of different galactose amounts are compared by overlay. C, Whole cell extracts were prepared from the same samples from A and analyzed by western blotting. Sld2 proteins and Orc6-FLAG proteins were detected with anti-Sld2 and anti-FLAG antibodies, respectively. *: non-specific background band.

To further explore how the protein level of Sld2-11D affects untimely replication, we controlled the protein level of Sld2-11D in *GALp-sld2-11D CDC45^JET1-1^* cells arrested in G1 phase with alpha factor by adding various concentrations of galactose to the medium and monitored DNA replication (Figure 4). The protein level of Sld2-11D increased as the galactose concentration increased (Figure 4B). The level of induced Sld2-11D protein was similar to endogenous Sld2, even after one hour treatment with 0.01% galactose, which was not enough to induce obvious DNA replication. However, the addition of more than 0.025% galactose induced DNA replication, and the induction of a higher protein level of Sld2-11D caused more DNA replication (Figure 4). Thus, the protein level of Sld2-11D is crucial for efficient induction of DNA replication.

### High Levels of Sld2 Cause Increased Gross Chromosome Rearrangements

**Figure 5:**
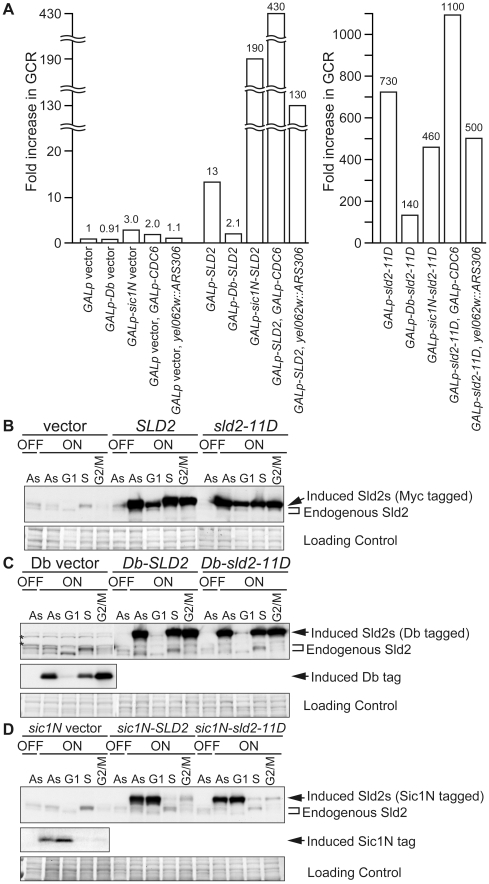
High-level expression of Sld2 in G1 induces GCR. A, Relative GCR values in Table 1 are graphically shown. The GCR rate of YST1007 (*GALp* vector) grown in galactose-containing medium is set as one. B, YST1007 (vector), YST1008 (*SLD2*) and YST1024 (*sld2-11D*) cells were grown in YPAD (OFF) or YPAGal (ON) medium (Asynchronous: As), and aliquots of the YPAGal culture were arrested in G1, S or G2/M phase with alpha factor, HU or nocodazole, respectively. Cells were collected, and Sld2 proteins were detected with anti-Sld2 antibody. The loading control shows the corresponding region of the Ponceau-S-stained membrane. C, YST1338 (Db vector), YST1062 (*Db-SLD2*) and YST1064 (*Db-sld2-11D*) cells were grown and analyzed as in B. The Db fragment (Myc-tagged) was expressed from the Db vector and detected with anti-Myc antibody. *: non-specific background bands. D, YST1159 (*sic1N* vector), YST1161 (*sic1N-SLD2*) and YST1162 (*sic1N-sld2-11D*) cells were grown and analyzed as in B. The Sic1N fragment (Myc-tagged) was expressed from the *sic1N* vector and detected with anti-Myc antibody.

The data shown above suggests that the Sld2 protein level in G1 phase is a limiting factor for the initiation of DNA replication. The limited level of Sld2 may contribute to the inhibition of untimely DNA initiation in G1 phase during normal cell proliferation. Because constitutive expression of Sld2 and Sld2-11D from the *GAL* promoter does not affect the overall rate of cell growth (data not shown), we hypothesized that this might cause a slight enhancement of initiation that does not confer slow cell growth or cell death. To detect such inefficient initiation, we employed the gross chromosome rearrangement (GCR) assay [24], which efficiently detects abnormal chromosomal transactions, such as untimely initiation. GCRs are chromosomal abnormalities, such as translocations, deletion of a chromosome arm, and interstitial deletions or inversions. In this assay, by measuring the loss rate of two counter-selectable markers, *URA3* and *CAN1* on chromosome V, the rate for GCR generation of the strain can be calculated [24]. When control cells harboring the empty *GALp* vector were grown in galactose-containing media, the GCR rate was 0.79×10^−10^/cell division (Table 1). When Sld2 was expressed, a GCR of 13 times higher was observed (Table 1, Figure 5A). This result suggests that high levels of Sld2 affect genome stability. Interestingly, more than 700 times higher GCR rate was observed when Sld2-11D was expressed (Table 1, Figure 5A). Because the expression level of Sld2 and Sld2-11D was similar (Figure 5B), the much higher GCR rate for Sld2-11D was possibly induced by the phosphomimetic property of the Sld2-11D protein. These results suggest that increased expression of Sld2 causes untimely initiation, and this occurs more frequently in Sld2-11D-expressing cells.

**Table 1:** The GCR rate for *GALp-SLD2* cells.

Strain	Genotype	GCR rate (×10^−10^/cell div.)		Relative value*1
		Glc (OFF)	Gal (ON)	
YST1007	*GALp* vector	1.5	0.79	1
YST1008	*GALp-SLD2*	0.70	11	13
YST1024	*GALp-sld2-11D*	1.7	580	730
YST1338	*GALp-Db*	0.84	0.72	0.91
YST1062	*GALp-Db-SLD2*	0.88	1.7	2.1
YST1064	*GALp-Db-sld2-11D*	1.1	110	140
YST1159	*GALp-sic1N*	0.75	2.3	3.0
YST1161	*GALp-sic1N-SLD2*	<1.9*2	150	190
YST1162	*GALp-sic1N-sld2-11D*	<1.4*2	370	460
YST1128	*GALp-CDC6 GALp* vector	ND*3	1.6	2.0
YST1129	*GALp-CDC6 GALp-SLD2*	<1.2*2	340	430
YST1130	*GALp-CDC6 GALp-sld2-11D*	2.3	870	1100
YST1743	*yel062w::ARS306 GALp* vector	1.8	0.89	1.1
YST1745	*yel062w::ARS306 GALp-SLD2*	<1.8*2	100	130
YST1744	*yel062w::ARS306 GALp-sld2-11D*	1.9	400	500
YST1051	*GALp-DPB11*	0.78	48	61

*1: The relative value of the GCR rate from the +Gal condition. The GCR rate of the control strain (YST1007) is set as 1.

*2: The number of plates on which mutants appeared was less than half (see Materials and Methods for details).

*3: The GCR rate could not be determined because colonies did not appear on all plates.

When untimely origin activation occurs through high levels of Sld2, re-assembly of the pre-RC is the next event required for re-replication to occur. Although the G1 phase is a window of time during which cells can form the pre-RC, the potential for pre-RC formation is limited by the fact that Cdc6 is unstable even during G1 [25]. Therefore, when untimely pre-RC activation is induced by high levels of Sld2 in the G1 phase, simultaneous expression of Cdc6 should cause a further increase in the GCR rate by increasing the potential for re-assembly of the pre-RC at activated origins. To test this idea, we combined *GALp-CDC6* and *GALp-SLD2* for simultaneous expression. In control cells, in which Cdc6 alone is expressed, the GCR rate changed very little (2.0 times higher than that of *GALp* vector). This result is expected because pre-RC assembly itself does not cause untimely activation of the pre-RC. In contrast, simultaneous expression of Cdc6 and Sld2 resulted in a highly elevated GCR rate. The GCR rate of *GALp-CDC6 GALp-SLD2* cells was 430 times higher than that of the control vector and was 31 times higher than that of *GALp-SLD2* cells (430×10^−10^/11×10^−10^ = 31), in which *SLD2* alone was expressed (Figure 5A and Table 1). Even with the *GALp-sld2-11D* background, enhancement of the GCR rate by simultaneous Cdc6 expression was observed (from 730 to 1100 times higher than control, Figure 5A and Table 1). These data further support the possibility that high levels of Sld2 in G1 phase cause untimely initiation.

### High-Level Sld2 during G1 Phase Is Responsible for the Elevated GCR Rate

To further investigate whether untimely initiation in G1 phase is the reason why high level of Sld2 induced a higher GCR rate, we modulated the Sld2 expression pattern during the cell cycle. For this purpose, two types of cell cycle-dependent degron tags were attached to the Sld2 N-terminus to control the accumulation pattern of Sld2 during the cell cycle. One tag was the destruction box (Db) of Clb2, which is unstable in G1 and is responsible for the degradation of Clb2 from late M to G1 phase [26]. The other tag was the N-terminal 100 amino acids of Sic1 (Sic1N), which is degraded when CDK is active [27]. Sic1N lacks the CDK inhibitory domain, and thus, its expression does not affect cell cycle progression ([28], data not shown).

When untagged Sld2s were expressed from the *GAL* promoter, their protein levels were high throughout the cell cycle (Figure 5B). In contrast, Db-tagged Sld2 specifically disappeared from G1 cell extracts, although it accumulated at a high level in S or G2/M extracts (Figure 5C). The GCR for the *GALp-Db-SLD2* strain was decreased to one-sixth of the *GALp-SLD2* strain (from 13- to 2.1-fold) and was only two-fold higher than that of the control *GALp-Db* strain (from 2.1- to 0.91-fold) (Table 1 and Figure 5A). The GCR rate of the *GALp-Db-sld2-11D* strain was also decreased to less than one-fifth of the *GALp-sld2-11D* strain (from 730- to 140-fold) (Table 1 and Figure 5A). When cells were not expressing Sld2, GCR rates did not increase in any case (Table 1 and Figure 5A). Therefore, destabilization of Sld2 in G1 phase by the Db-tag suppressed the increase in GCR rates in *GALp-SLD2* cells.

On the contrary, Sic1N-tagged Sld2 was destabilized specifically from S to M phase, and importantly, its protein level was high in G1 phase (Figure 5D). The GCR rate of the *GALp-sic1N-SLD2* strain did not decrease, but rather, it increased from 13- to 190-fold (Table 1 and Figure 5A), although the exact reason for this increase is not clear. The GCR rate of the *GALp-sic1N-sld2-11D* strain was only affected modestly by protein destabilization from S to M phase (reduced from 730- to 460-fold) (Table 1 and Figure 5A). Overall, these data indicate that high level of Sld2 in G1 phase is the primary reason for the elevated GCR.

### The GCR in High-Level Sld2 Cells Is Enhanced by Replication Origin Insertion

As described above, high levels of Sld2 increase the GCR rate, and the effect is enhanced by simultaneous expression of Cdc6. These results strongly suggest that untimely replication in G1 induced by high level of Sld2 is the primary reason for the elevated GCR. However, another mechanism is possible that high level of Sld2 in G1 might titer away other replication factors and compromise either pre-RC formation or fork progression, which would then cause the GCRs, although this mechanism is not very likely because there are no known roles of Sld2 in pre-RC assembly and fork elongation. To test the possibility, we inserted the efficient replication origin *ARS306* at the *YEL062w* locus, which is proximal to the GCR marker *CAN1* (*YEL063c*) on chromosome V. We deleted original *ARS306* on chromosome III to avoid the duplication of the *ARS306* sequence over two different chromosomes. If elevated GCR in *GALp-SLD2* cells is caused by repetitive origin firing, *yel062w::ARS306* cells would show a higher GCR rate, while defects in pre-RC assembly or fork progression would be rescued by ARS insertion and GCR would be repressed. In the control (*GALp* vector *yel062w::ARS306*) cells, *ARS306* insertion did not affect the GCR rate (Table 1 and Figure 5A). In contrast, the insertion increased the GCR rate of *GALp-SLD2* cells approximately ten fold when Sld2 was induced (from 13- to 130-fold), although *GALp-sld2-11D* cells did not show a significant change (from 730- to 500-fold) (Table 1 and Figure 5A). These results exclude the possibility that high level of Sld2 causes defective origin activation or fork progression.

### Excess Sld2-11D Expression Similar to the Endogenous Level Can Induce GCR

**Figure 6:**
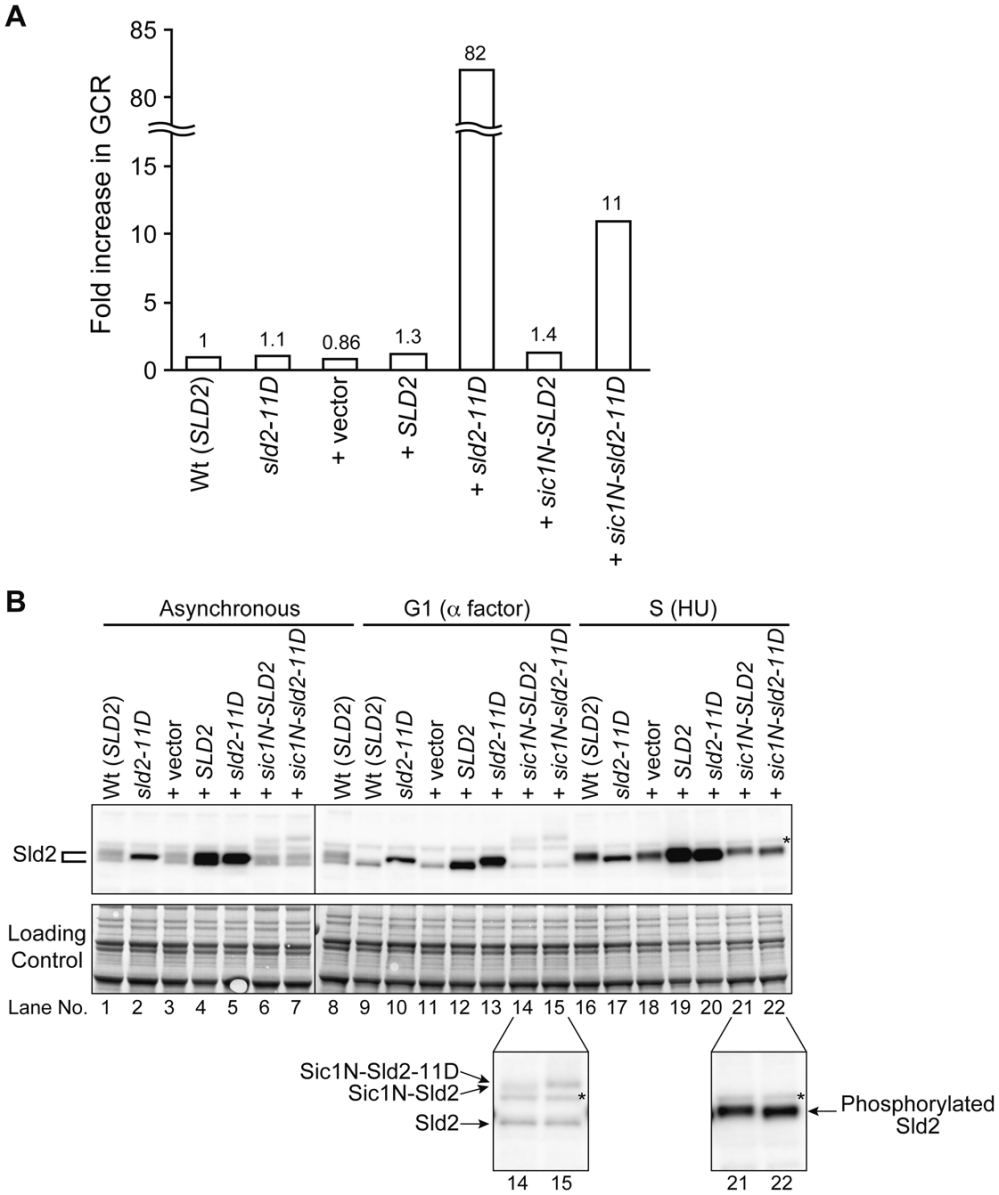
Extra Sld2-11D expression similar at endogenous level can induce GCR. A, Relative GCR values in Table 2 are graphically shown. The GCR rate of RDKY3615 (Wt (*SLD2*)) is set as one. B, RDKY3615 (Wt (*SLD2*)), YST1336 (*sld2-11D*), YST1090 (+vector), YST1737 (+*SLD2*), YST1739 (+*sld2-11D*), YST1747 (+*sic1N-SLD2*) and YST1749 (*sic1N-sld2-11D*) cells were grown (asynchronous), and aliquots of the culture were arrested in G1 or S phase with alpha factor or HU, respectively. Whole cell extracts were prepared and subjected to western blotting. Sld2 proteins were detected with anti-Sld2 antibody. The loading control shows the corresponding region of the Ponceau-S-stained membrane. *: non-specific background band. Lanes 14, 15, 21 and 22 are enlarged to show the Sic1N-Sld2 bands (bottom).

As shown in Figure 2 and Figure 3, in the *CDC45^JET1-1^* background, untimely DNA replication cannot be induced by G1-level Sld2-11D. This result suggests that even Sld2-11D would not affect the overall genome stability, such as the GCR rate, if it is expressed at an endogenous level. As expected, when Sld2-11D was expressed as a sole genomic copy of Sld2 by replacing the genomic copy of *SLD2* with *sld2-11D*, the GCR rate was almost the same as that of the wild type (Figure 6A and Table 2). The Sld2-11D protein level in G1 was higher than that of the wild type in G1 but lower than that of the wild type in S phase (Figure 6B, compare lanes 9, 10, and 16).

**Table 2:** The GCR rate of cells with extra copies of *SLD2*.

Strain	Genotype	GCR rate (×10^−10^/cell div.)	Relative value*1
RDKY3615	*SLD2* (wt) control	1.8	1
YST1336	*sld2-11D*	1.9	1.1
YST1090	*SLD2* (wt)+YIp vector	1.5	0.86
YST1737	*SLD2* (wt)+YIp-*SLD2*	2.2	1.3
YST1739	*SLD2* (wt)+YIp-*sld2-11D*	150	82
YST1747	*SLD2* (wt)+YIp-*sic1N-SLD2*	2.4	1.4
YST1749	*SLD2* (wt)+YIp-*sic1N-sld2-11D*	20	11

*1: The relative value of the GCR rate. The GCR rate of control strain (RDKY3615) is set as 1.

In contrast, because S phase-level Sld2-11D can induce untimely DNA replication in G1/S phase-arrested cells (Figure 2 and Figure 3), we asked whether the S phase-level Sld2-11D in G1 phase can induce a higher GCR rate or not. For this purpose, *SLD2* promoter-regulated *SLD2*, *sld2-11D*, *sic1N-SLD2*, or *sic1N-sld2-11D* were inserted at the *LEU2* locus. Cells with extra copies with *SLD2* or *sld2-11D* accumulated Sld2 or Sld2-11D protein at a level higher than that of wild type in G1 and is similar to that in S phase (Figure 6B, compare lanes 9, 11–13, 16, and 18). When Sic1N-Sld2 or Sic1N-Sld2-11D was expressed, they were observed only in G1 cells (Figure 6B lanes 14, 15, 21 and 22, and data not shown). Therefore, Sld2 amount was increased only in G1 phase in these cells. The GCR rate was increased when cells had an excess amount of Sld2-11D (+*sld2-11D* cells: 82-fold, +*sic1N-sld2-11D* cells: 11-fold (Figure 6A and Table 2)), and even the cells with an excess amount of Sld2 tended to increase GCR (+*Sld2* cells: 1.3-fold, +*sic1N-Sld2* cells: 1.4-fold (Figure 6A and Table 2)). These data indicate that it is important to keep the Sld2 level low to prevent untimely replication in G1 cells.

### The Protein Level of Other Initiation Factors Also Contributes to Stable Genome Maintenance

In addition to Sld2, Dpb11 and Sld3 are required for initiation, and these proteins all form a complex regulated by CDK, which is crucial for the initiation of DNA replication. The expression of Dpb11 and Sld3 are constant throughout the cell cycle, and their expression levels are relatively low ([29], data not shown). This fact also raises the possibility that high-level expression of Sld3 or Dpb11 might cause untimely initiation and hence a higher GCR rate. We tested this possibility with a *GALp-DPB11* strain. A High level of expression of Dpb11 resulted in an approximately 60 times higher GCR rate (Table 1). This result suggests that limiting the level of proteins involved in the initiation reaction is important to prevent untimely initiation and hence is important for genome stability.

### Untimely Initiation Causes Double-Strand DNA Breaks and Checkpoint Activation Later in the Cell Cycle

**Figure 7:**
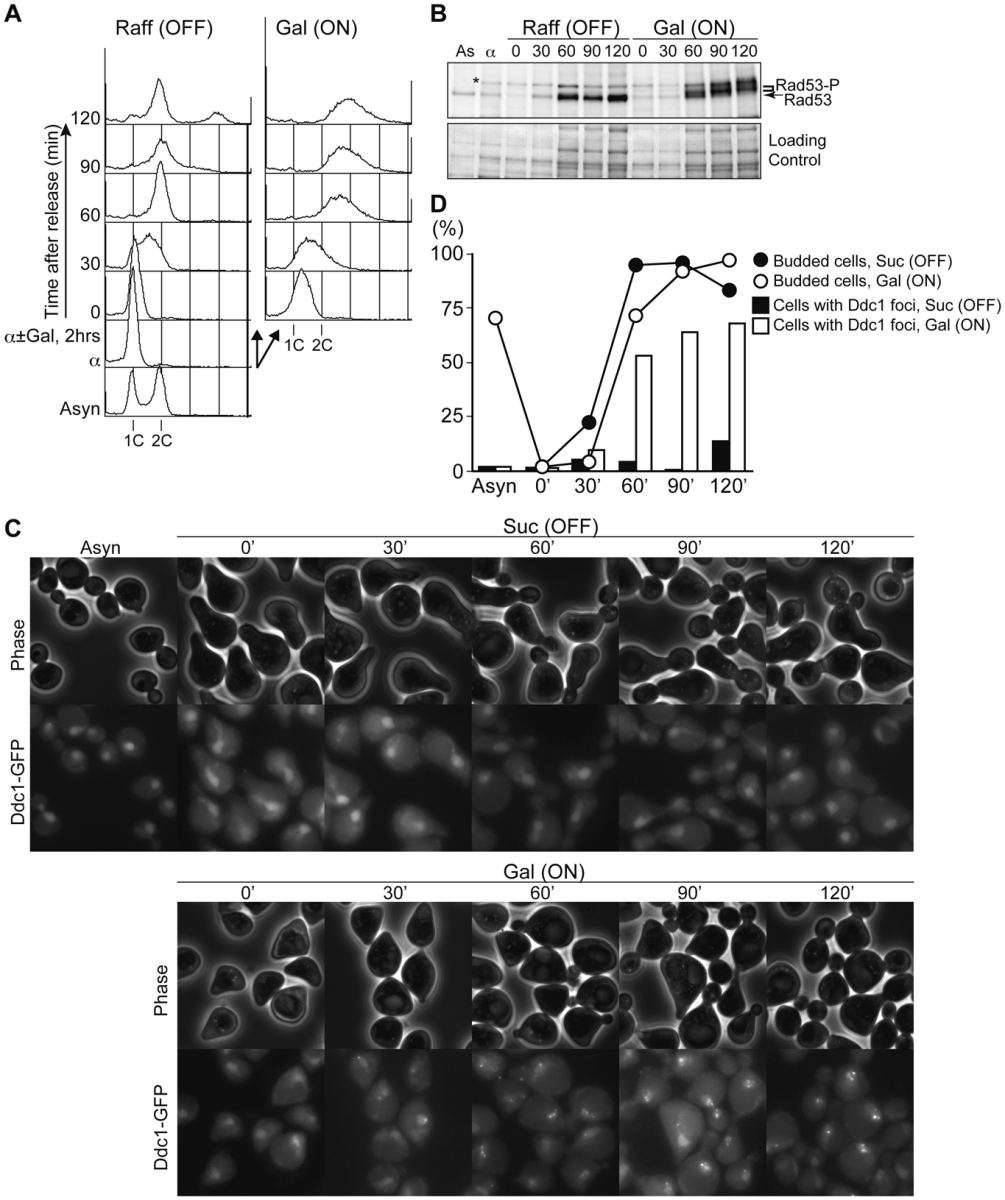
Untimely DNA replication conferred the accumulation of phosphorylated Rad53 and Ddc1 foci after S phase. A, YST1700 cells (*CDC45^JET1-1^ GALp-sld2-11D DDC1-GFP*) were grown in YPARaffinose medium (Asyn) and arrested in G1 phase with alpha factor (α). The culture was then split into two portions, and galactose was added to one portion (Gal (ON)) and further incubated for 2 hours. Then cells were released into fresh YPAD, and samples were taken at the indicated times (0–120 min). The DNA contents of the samples were analyzed by flow cytometry. B, Whole cell extracts were prepared from the same samples from A and subjected to western blotting. Rad53 protein was detected with anti-Rad53 antibody. *: non-specific background band. C, D, YST1700 cells were grown in SC-Sucrose medium, arrested in G1 and split into two portions, and galactose was added to one portion (Gal (ON)) and released into fresh SC-glucose. Samples were taken at the indicated times (0–120 min), and Ddc1-GFP was observed under the microscope (C). The proportion of cells with Ddc1-foci or bud was counted (D).

As shown in Figure 1, re-replication induced by untimely DNA replication in G1 causes abnormal chromosome composition. Relicensing experiments using *Xenopus* egg extracts suggested that multiple rounds of initiation from the same origin would generate consecutive replication forks travelling in the same direction and that they may finally collide [30]. Such collisions may generate extruded DNA strands, which will be recognized by DNA damage response machinery [30], [31]. A similar situation would occur in re-replicating DNA induced by untimely initiation in G1. To address this possibility, we have monitored the phosphorylation of Rad53, an essential protein kinase required for cell cycle checkpoint function, and foci formation of Ddc1, a subunit of a PCNA-like complex required for DNA damage response (Figure 7). *CDC45^JET1-1^ GALp-sld2-11D DDC1-GFP* cells were arrested in G1 phase with alpha factor, Sld2-11D was expressed temporally by galactose addition, and then cells were synchronously released into glucose-containing medium. When Sld2-11D is not expressed [Raff (OFF)], cells can finish the cell cycle and enter into the next cell cycle after the release (Figure 7A). In contrast, when Sld2-11D was induced [Gal (ON)], untimely DNA replication occurred as in previous experiments (see Figure S1, S2, S3). Release from G1 arrest allowed cells to enter S phase within 30 min. After bulk DNA replication, cells were arrested in G2 with more than 2C DNA (Figure 7A). As cells pass through S phase, phosphorylated Rad53 and Ddc1 foci accumulated only when Sld2-11D was induced (Figure 7B–7D). This result indicates that a checkpoint pathway is activated in these cells and further suggests the occurrence DNA damages. Because Ddc1-foci formation is observed in many cells, this indicates that at least double-strand DNA breaks were generated as cells go through S phase.

## Discussion

Because DNA replication in eukaryotes occurs as a two-step reaction, activities for these steps, pre-RC assembly and activation, must be separated to prevent re-replication in the cell cycle. In this study, we have investigated how untimely activation of the pre-RC, the initiation of DNA replication, is prevented in G1 phase. When untimely pre-RC activation and consequent DNA replication are induced in G1-arrested cells, cells lose viability very quickly (Figure S1). Because the induction of untimely initiation of DNA replication itself does not immediately activate Rad53, a checkpoint kinase activated by DNA damage (Figure 7, data not shown), it is likely that abnormal chromosome structures and/or DNA damage generated later by re-replication are genotoxic rather than untimely initiation itself. In fact, the survivors recovered from re-replicating cells frequently have abnormal chromosome compositions (Figure 1). Although at least double-strand DNA breaks are occurring later in the cell cycle (Figure 7) as in the case of abrogation of the mechanism to inhibit relicensing [32], it is still unclear what types of structures cause such damage or whether other types of DNA damage are generated by this re-replication. Multiple rounds of initiation may generate multiple replication forks chasing one another along the same DNA template. For example, head-to-tail replication fork collision is suggested to occur when re-replication is induced by relicensing of activated origins in *Xenopus* egg extracts [30].

When the mechanism to inhibit relicensing is partially abrogated in *Saccharomyces cerevisiae*, specific chromosomal loci are preferentially re-replicated and potentially induce gene amplification [33], [34]. In Figure 1, three independent survivors showed that similar chromosome rearrangement, and this may suggest the existence of hot spot(s) for chromosome rearrangement (Figure 1A, lane 8–10). This site is different from that preferentially re-replicated when relicensing inhibition is abrogated. This difference might be caused by the difference in the order of activation of origins. When untimely initiation in G1 phase is induced, the temporal control of replication origins observed in the normal S phase is likely to be maintained [13], while re-replication in G2/M phase caused by relicensing primarily occurs at a subset of both active and latent origins [34]. Therefore, it would be intriguing to determine whether hotspots for chromosome rearrangement appear when untimely initiation is induced. It would also be interesting to analyze the chromosome context surrounding if such hot spots exist. Moreover, whole chromosome duplication is observed very frequently in survivors (Figure 1). The reason for this finding also should be addressed in a future study to understand the impact of untimely DNA replication on genome stability.

We have shown that the regulation of the protein-level of initiation factors such as Sld2 and Dpb11 is important to prevent untimely initiation in G1 phase, in addition to the previously described context of CDK phosphorylation of Sld2 and Sld3. The high-level expression of Sld2 or Dpb11 alone resulted in a higher GCR rate (Figure 5 and Table 1). This result is the first example indicating that the protein-level of initiation factors directly affects genome stability and further confirms the direct relationship between the G1/S regulatory machinery and genome stability, both of which are frequently deregulated in human cancer cells [31], [35]–[40]. In budding yeast cells, the protein levels of replication factors required for initiation, such as Sld3, Dpb11, Cdc45, Mcm2-7 and GINS, are constant throughout the cell cycle and only the protein level of Sld2 fluctuates ([15], [41]–[43], our unpublished data). Of these replication factors, Sld2, Sld3 and Dpb11 are only required for the initiation step. Although phosphorylation of Sld2 by S-CDK greatly enhances the interaction between Sld2 and Dpb11, in vitro analysis suggests that unphosphorylated Sld2 and Dpb11 can interact, although the interaction is inefficient [15], [16]. Because G1-level Sld2-11D is not sufficient to induce DNA replication but G1/S-level Sld2 is (Figure 2 and Figure 3), cell cycle-regulated Sld2 expression is important not only for the enhancement of initiation in S phase but also for the prevention of unfavorable Sld2-Dpb11 complex formation in G1 phase to inhibit untimely initiation. The concentration of other key initiation factors, Dpb11 and Sld3, is relatively low throughout the cell cycle (Tanaka and Araki, manuscripts in preparation), and this must also be important to prevent untimely initiation because a high level of expression of Dpb11 resulted in a higher GCR rate.

It is known that deletions of or defects in factors that play a role in the DNA damage response pathway elevate GCR in budding yeast [44]–[46]. Dpb11 is known to play a role in the intra S phase and DNA damage checkpoint pathway [29], [47]. Because Sld2 interacts with Dbp11, a high level of Sld2 could disturb Dpb11's checkpoint function and result in increased GCR, although this seems unlikely. In budding yeast, DNA damage is mainly recognized in S and G2 phase, and cells with DNA damage do not show a remarkable delay for S phase entry [48]. If a high level of Sld2 affects checkpoint function, a high level of Sld2 in S to M phase should cause elevated GCR, and a high level of Sld2 in G1 would not affect GCR generation. However, our results showed the opposite effect, which indicates that the higher GCR rate observed in Sld2-expressing cells is not caused by checkpoint failure.

Our results indicate that multiple regulatory mechanisms are employed to prevent untimely initiation in G1. At least three mechanisms, the hypophosphorylated status of essential initiation proteins Sld2 and Sld3 and the low level of Sld2, contribute to prevent untimely activation of replication origins. These mechanisms are independent; therefore, disruption of one regulatory mechanism, for example, the high level of expression of Sld2, is enough to elevate GCR, and the simultaneous deregulation of these mechanisms causes more severe phenotypes. For example, a high level of expression of Sld2-11D (both the protein level and phosphorylation of Sld2 are deregulated) resulted in a very high GCR rate (Table 1 and Figure 5), and the combination of *CDC45^JET1-1^* and *sld2-11D* (both of the phosphorylations of Sld2 and Sld3 are bypassed) frequently generated chromosome rearrangements and aneuploidy (Figure S5). When all of the regulatory mechanisms are bypassed in *CDC45^JET1-1^ GALp-SLD2-D* cells, cells die (Figure S1 and Figure 2A). Although each inhibitory mechanism can mostly block the initiation of DNA replication, each of them alone is not sufficient. Therefore, multiple mechanisms are important to the robustness of the system to prevent untimely initiation and hence for stable genome maintenance over generations.

Although budding yeast is so far the only system in which the untimely initiation of DNA replication can be artificially induced [13], [14], regulation of initiation by combining CDK phosphorylation and dosage control of initiation factors might also be employed in other eukaryotes. The CDK requirement for the initiation of DNA replication is a highly conserved feature in eukaryotes. In vertebrates, TopBP1/Cut5/Mus101 and RecQL4 are thought to be the orthologues of Dpb11 and Sld2, respectively, because of sequence similarities and their roles in DNA replication (reviewed in [3], [49]). In *Xenopus* egg extracts, both Cut5 and RecQL4 bind chromatin before initiation and interact each other, and the Sld2-homology domain of RecQL4 contains many potential CDK phosphorylation sites [50]–[53], although whether or not phosphorylation of RecQL4 is required for these functions is unclear. Recently, novel factors called Treslin/Ticrr, GemC1 and DUE-B were reported as essential factors for initiation [54]–[57]. Treslin is distantly related to Sld3 [58], and like budding yeast Sld3, phosphorylated Treslin interacts with N-terminal tandem BRCTs of TopBP1 in *Xenopus* egg extracts [56]. GemC1 also has multiple CDK phosphorylation sites that are important for the initiation of DNA replication and interacts with TopBP1 [54]. Therefore, these proteins are possible functional analogues of Sld3. Of these orthologues/analogues of Sld2, Sld3 and Dpb11, the expression of TopBP1 is under the control of E2F, a G1-S specific transcription factor [59].

Genomic instability is a hallmark of human cancer cells [35], [38]. Based on our results, the deregulation of initiation factors in human cells may be important in the induction of genomic instability and cancer. The retinoblastoma/E2F pathway is known to be deregulated frequently in cancer cells [60], [61]. As described above, TopBP1 is one of its targets, although the expression level of TopBP1 has not been precisely determined. Interestingly, in osteosarcoma, chromosomal rearrangements and genomic imbalances affecting 8q24 in which the RECQL4 gene maps are frequent and the increased expression of RecQL4 are correlated to some type of chromosome instability [62]. Therefore, it is possible that untimely initiation is occurring in these cells by the high-level expression of initiation factors, perhaps triggering genomic instability. In summary, our data in budding yeast provide a good model to understand how untimely initiation is prevented and hence how stable genome maintenance is achieved in eukaryotic cells.

## Materials and Methods

### Strains and Media

The strains used in this study are listed in Table 3. All strains used in the GCR assay are derived from RDKY3615. All others are derived from W303-1a. Cells were grown in rich medium YPA (1% yeast extract, 2% Bacto-peptone and 40 µg/ml adenine) or Synthetic Complete (SC: 0.67% Yeast Nitrogen Base, supplemented with amino acids) supplemented with 2% sugar (glucose, galactose, raffinose, or sucrose). Cells were arrested in G1 with 30 (if release was required) or 100 ng/ml alpha factor for *Δbar1* strains or 10 µg/ml alpha factor for *BAR1* (wild type) strains. For the cell cycle block in S and G2/M phase, 200 mM hydroxyurea (HU) and 5 µg/ml nocodazole were added to the medium.

**Table 3:** Strains.

Name	Genotype	Background or Reference
W303-1a *Δbar1*	*MATa ade2-1 ura3-1 his3-11,15 trp1-1 leu2-3,112 can1-100 Δbar1*	Laboratory stock
YST556	*CDC45^JET1-1^*	W303-1a *Δbar1*
YST559	*CDC45^JET1-1^ ura3-1::GALp-MycHis_9_ (URA3)*×2 copies	W303-1a *Δbar1*
YST560	*CDC45^JET1-1^ ura3-1::GALp-SLD2-MycHis_9_ (URA3)*	W303-1a *Δbar1*
YST561	*CDC45^JET1-1^ ura3-1::GALp-SLD2-MycHis_9_ (URA3)*×2 copies	W303-1a *Δbar1*
YST562	*CDC45^JET1-1^ ura3-1::GALp-sld2-11D-MycHis_9_ (URA3)*	W303-1a *Δbar1*
YST563	*CDC45^JET1-1^ ura3-1::GALp-sld2-11D-MycHis_9_ (URA3)*×2 copies	W303-1a *Δbar1*
YST573	*CDC45^JET1-1^ ura3-1::GALp-sld2-11D-MycHis_9_ (URA3) leu2-3,112::GALp-MycHis_9_ (LEU2)*×2 copies	W303-1a *Δbar1*
YST575	*CDC45^JET1-1^ ura3-1::GALp-sld2-11D-MycHis_9_ (URA3) leu2-3,112::GALp-DBF4-MycHis_9_ (LEU2)*×2 copies	W303-1a *Δbar1*
YST615	*CDC45^JET1-1^ ura3-1::GALp-sld2-T84D-MycHis_9_ (URA3)*×2 copies	W303-1a *Δbar1*
YST631	*CDC45^JET1-1^ cdc7-4 ura3-1::GALp-sld2-11D-MycHis_9_ (URA3) DBF4-MycHis_9_::TetO-DBF4::kanMX CDT1-GFP::kanMX*	W303-1a *Δbar1*
YST816	*CDC45^JET1-1^ sld2-11D::kanMX*	W303-1a *Δbar1*
YST819	*CDC45^JET1-1^ sld2-11D::kanMX* (re-streak of YST816)	W303-1a *Δbar1*
YST820	*CDC45^JET1-1^ sld2-11D::kanMX*	W303-1a *Δbar1*
YST821	*CDC45^JET1-1^ sld2-11D::kanMX*	W303-1a *Δbar1*
YST827	*CDC45^JET1-1^ his3-11,15::GALp-sic1ΔNT-MycHis_9_ (HIS3)*	W303-1a *Δbar1*
YST829	*CDC45^JET1-1^ his3-11,15::GALp-sic1ΔNT-MycHis_9_ (HIS3) sld2-11D::kanMX*	W303-1a *Δbar1*
YST831	*sld2-11D::kanMX*	W303-1a *Δbar1*
YST1332	*ura3-1::GALp-sic1ΔNT-MycHis_9_ (URA3)*	W303-1a *Δbar1*
YST1447	*CDC45^JET1-1^ ura3-1::GALp-sld2-11D-MycHis_9_ (URA3) DBF4-MycHis_9_::TetO-DBF4::kanMX CDT1-GFP::kanMX*	W303-1a *Δbar1*
YST1698	*CDC45^JET1-1^ ura3-1::GALp-sld2-11D-MycHis_9_ (URA3)*×2 copies *ORC6-3xFLAG-1xHA::kanMX*	W303-1a *Δbar1*
YST1700	*CDC45^JET1-1^ ura3-1::GALp-sld2-11D-MycHis_9_ (URA3)*×2 copies *DDC1-GFP::kanMX*	W303-1a *Δbar1*
RDKY3615	*MATa ade2Δ1 ade8 ura3-52 his3Δ200 hom3-10 leu2Δ1 lys2ΔBgl trp1Δ63 hxt13/yel069w::URA3*	Chen & Kolodner (1999)
YST1007	*leu2Δ1::GALp-MycHis_9_ (LEU2)*	RDKY3615
YST1008	*leu2Δ1::GALp-SLD2-MycHis_9_ (LEU2)*	RDKY3615
YST1024	*leu2Δ1::GALp-sld2-11D-MycHis_9_ (LEU2)*	RDKY3615
YST1338	*leu2Δ1::GALp-clb2 destruction box (Db)-MycHis_9_ (LEU2)*	RDKY3615
YST1062	*leu2Δ1::GALp-clb2 destruction box (Db)-SLD2-MycHis_9_ (LEU2)*	RDKY3615
YST1064	*leu2Δ1::GALp-clb2 destruction box (Db)- sld2-11D -MycHis_9_ (LEU2)*	RDKY3615
YST1159	*leu2Δ1::GALp-sic1N100-MycHis_9_ (LEU2)*	RDKY3615
YST1161	*leu2Δ1::GALp-sic1N100-SLD2-MycHis_9_ (LEU2)*	RDKY3615
YST1162	*leu2Δ1::GALp-sic1N100-sld2-11D-MycHis_9_ (LEU2)*	RDKY3615
YST1128	*leu2Δ1::GALp-MycHis_9_ (LEU2) CDC6::GALp-CDC6 (kanMX, HIS3)*	RDKY3615
YST1129	*leu2Δ1::GALp-SLD2-MycHis_9_ (LEU2) CDC6::GALp-CDC6 (kanMX, HIS3)*	RDKY3615
YST1130	*leu2Δ1::GALp-sld2-11D-MycHis_9_ (LEU2) CDC6::GALp-CDC6 (kanMX, HIS3)*	RDKY3615
YST1743	*yel062w::ARS306 ars306::nat1 leu2Δ1::GALp-MycHis_9_ (LEU2)*	RDKY3615
YST1745	*yel062w::ARS306 ars306::nat1 leu2Δ1::GALp-SLD2-MycHis_9_ (LEU2)*	RDKY3615
YST1744	*yel062w::ARS306 ars306::nat1 leu2Δ1::GALp-sld2-11D-MycHis_9_ (LEU2)*	RDKY3615
YST1051	*leu2Δ1::GALp-DPB11 (LEU2)*	RDKY3615
YST1336	*sld2-11D*	RDKY3615
YST1090	*leu2Δ1::LEU2*	RDKY3615
YST1737	*leu2Δ1::SLD2 (LEU2)*×3 copies	RDKY3615
YST1739	*leu2Δ1::sld2-11D (LEU2)*×2 copies	RDKY3615
YST1747	*leu2Δ1::sic1N100-SLD2 (LEU2)*×2 copies	RDKY3615
YST1749	*leu2Δ1::sic1N100-sld2-11D (LEU2)*×2 copies	RDKY3615

### Immunoblotting and Flow Cytometry

Endogenous Sld2 and Myc-tagged Sld2 were detected with anti-Sld2 polyclonal antibodies [16]. Orc6 was detected with the SB49 monoclonal antibody [63]. FLAG-tagged Orc6 was detected with the M2 monoclonal antibody (Sigma). Myc-tagged Sld2-11D, Db-tag and Sic1-tag were detected with the 9E10 monoclonal antibody. Rad53 was detected with the anti-Rad53 serum [48]. Flow cytometry was performed as described elsewhere [11]. To quantify the increase in DNA content, the average of DNA contents was calculated with the CellQuestPro program (Beckton-Dickinson).

### Pulsed-Field Gel Electrophoresis and Band Quantification

Yeast chromosomes were separated with the CHEF-DRII (Bio-Rad) in a 0.8% agarose gel with 0.5× TBE buffer. Gel images were acquired and analyzed with the LAS-4000 mini and the Multi Gauge software (GE Healthcare).

### The GCR Assay

The GCR assay was performed as previously described [24], [64]. Briefly, in the typical experiment, five independent colonies were grown in the appropriate medium and then spread onto synthetic medium containing canavanine (CAN) and 5-fluoroorotic acid (5FOA). The number of colonies formed on CAN+5FOA plates was counted, and the median was used to calculate the GCR rate. In the case of *2 in Table 1, colonies appeared on less than the half of plates, and the GCR rate was calculated from the highest colony number.

## Supporting Information

Figure S1 Untimely initiation of DNA replication in G1 is highly toxic to cells. A, YST559 (*JET1-1 GALp* vector), YST561 (*JET1-1 GALp-SLD2*), YST563 (*JET1-1 GALp-sld2-11D*) and YST615 (*JET1-1 GALp-sld2-T84D*) cells were grown in YPA raffinose medium (Asyn) and arrested in G1 phase with alpha factor, glucose (Glc (OFF)) or galactose (Gal (ON)) was added and samples were taken at the indicated times. The DNA contents of the samples were analyzed by flow cytometry. B, Small aliquots of the same samples from A were taken and spread onto YPAD plates, and the viability was calculated from the number of colonies that appeared on the plate after incubation. C, Whole cell extracts were prepared from the same samples as A and analyzed by western blotting. Sld2 proteins and Orc6 protein were detected with anti-Sld2 and anti-Orc6 antibodies, respectively. The loading control shows the corresponding region of the Ponceau-S-stained membrane. *: non-specific background band.
(TIF)

Figure S2 *cdc7-4* blocks untimely initiation of DNA replication in G1 and rescues cells from lethality. A, YST1447 (*CDC7* (wt)) and YST631 (*cdc7-4*) cells were grown in YPARaffinose medium at 25°C (Asyn) and arrested in G1 phase with α factor (α arrest). The culture was then split into two portions. One portion was shifted to 37°C, and the other was kept at 25°C. After 15 minutes of incubation, each culture was split into two portions again. Galactose was added to one portion (shown as Gal (ON)), and incubation was continued. Samples were taken at the indicated times (0–4 hours) and were analyzed by flow cytometry. B, Small aliquots of the same samples in A were taken and incubated in YPAD containing α factor for 60 minutes at 25°C. Next, the viability was measured as in Figure S1B. C, Whole cell extracts were prepared using the same samples in A and were analyzed by western blotting. Sld2 proteins were detected with anti-Sld2 antibody. The loading control shows the corresponding region of the Ponceau-S-stained membrane. *: non-specific background band.
(TIF)

Figure S3 Dbf4 expression enhances the untimely initiation of DNA replication in G1. A, YST573 (*JET1 GALp-sld2-11D GALp* vector) and YST575 (*JET1 GALp-sld2-11D GALp-DBF4*) cells were grown in YPARaffinose medium at 25°C (Asyn) and arrested in G1 phase with alpha factor, and the culture was split into two portions. Galactose was added to one portion (Gal (ON)), and the incubation was continued. Samples were taken at the indicated times (0–4 hours) and analyzed by flow cytometry. B, An overlay of the flow cytometry profiles of A. C, Small aliquots of the same samples in A were taken, and the viability was measured as in Figure S1B. D, Proportion of cells with buds. C, Whole cell extracts were prepared from the same samples in A and were analyzed by western blotting. Sld2s, Orc6 and Myc-tagged Dbf4 proteins were detected with anti-Sld2, anti-Orc6 and anti-Myc antibodies, respectively. The loading control shows the corresponding region of the Ponceau-S-stained membrane.
(TIF)

Figure S4 Control pulsed-field gel electrophoresis data for Figure 1. A, Chromosomal DNA from wild-type W303-1a *Δbar1* colonies (WT survivors 1–10) that appeared on the YPAD plate was analyzed with pulsed-field gel electrophoresis. Before plating, cells were grown as in Figure S1A with (α+Galactose, 1 hr) or without (α w/o Galactose, 1 hr) galactose. The same cells were grown in YPAD and analyzed as a control (lane C). Quantified profiles for each lane are shown in bottom. B, The chromosomal DNA of the YST563 (*JET1-1 GALp-sld2-11D*) colonies (survivors 1–8) that appeared on YPAD plates after glucose incubation (Glc (OFF))) in Figure S1 was analyzed. Abnormal chromosome bands are indicated with arrowheads.
(TIF)

Figure S5 Chromosomes are unstable in *CDC45^JET1-1^ sld2-11D* cells. A, Chromosomal DNA from YST556 (*JET1-1*) YST819 (*JET1-1 sld2-11D*, #1), YST820 (*JET1-1 sld2-11D*, #2) and YST821 (*JET1-1 sld2-11D*, #3) was analyzed with pulsed-field gel electrophoresis. The same sample was loaded in duplicate for each strain. Abnormal chromosome bands are indicated with arrowheads. B, The profiles of the band intensities of A are shown. Abnormal chromosome bands are indicated with arrowheads.
(TIF)
