# Supplementary figures and images for: Multiple Regulatory Mechanisms to Inhibit Untimely Initiation of DNA Replication Are Important for Stable Genome Maintenance
Source: PLoS Genet. 2011 Jun 16;7(6):e1002136. doi: 10.1371/journal.pgen.1002136 (PMC3116906; doi:10.1371/journal.pgen.1002136)

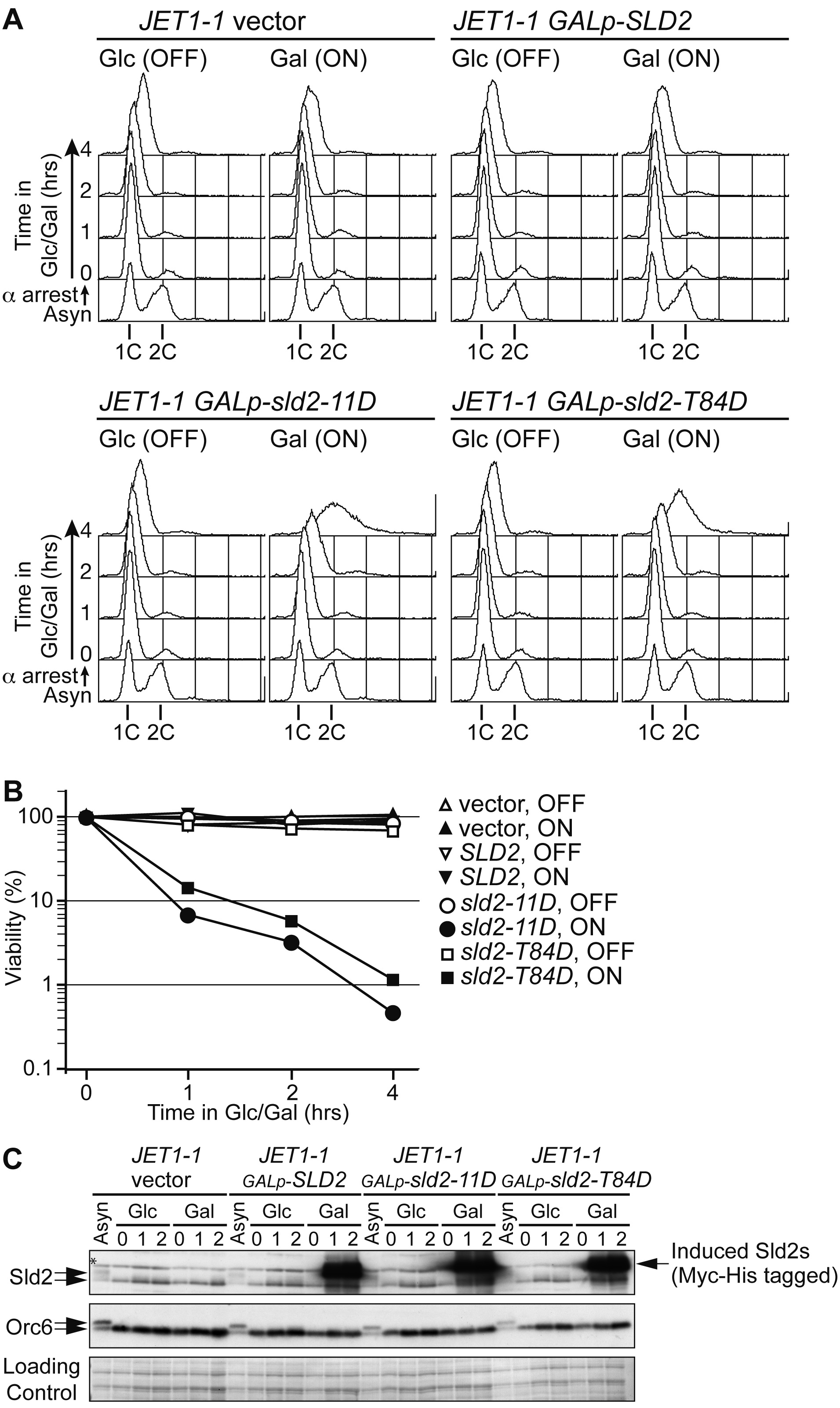

Supplement: Figure S1 — Untimely initiation of DNA replication in G1 is highly toxic to cells. A, YST559 (JET1-1 GALp vector), YST561 (JET1-1 GALp-SLD2), YST563 (JET1-1 GALp-sld2-11D) and YST615 (JET1-1 GALp-sld2-T84D) cells were grown in YPA raffinose medium (Asyn) and arrested in G1 phase with alpha factor, glucose (Glc (OFF)) or galactose (Gal (ON)) was added and samples were taken at the indicated times. The DNA contents of the samples were analyzed by flow cytometry. B, Small aliquots of the same samples from A were taken and spread onto YPAD plates, and the viability was calculated from the number of colonies that appeared on the plate after incubation. C, Whole cell extracts were prepared from the same samples as A and analyzed by western blotting. Sld2 proteins and Orc6 protein were detected with anti-Sld2 and anti-Orc6 antibodies, respectively. The loading control shows the corresponding region of the Ponceau-S-stained membrane. *: non-specific background band. (TIF) [file pgen.1002136.s001.tif]

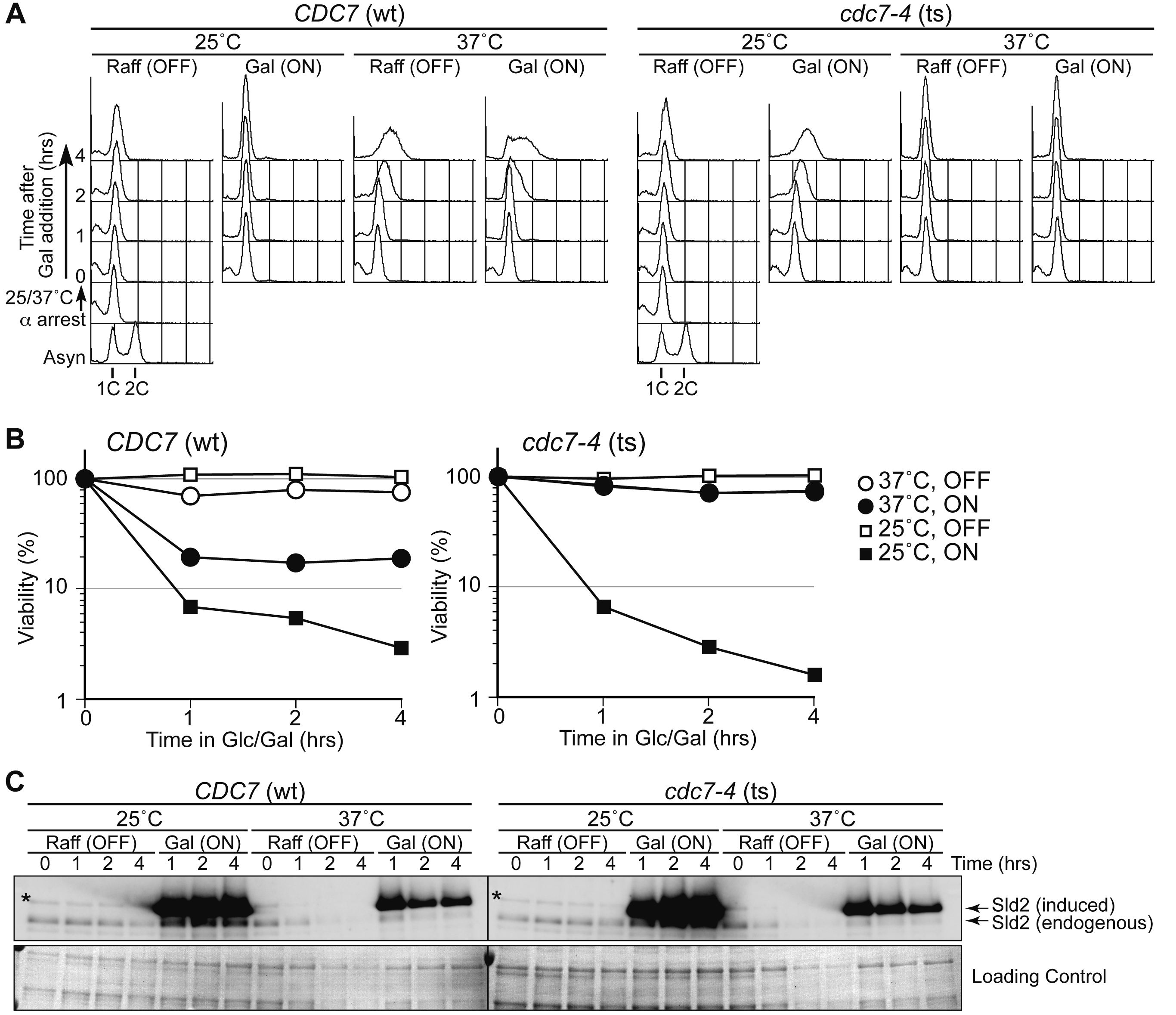

Supplement: Figure S2 — cdc7-4 blocks untimely initiation of DNA replication in G1 and rescues cells from lethality. A, YST1447 (CDC7 (wt)) and YST631 (cdc7-4) cells were grown in YPARaffinose medium at 25°C (Asyn) and arrested in G1 phase with α factor (α arrest). The culture was then split into two portions. One portion was shifted to 37°C, and the other was kept at 25°C. After 15 minutes of incubation, each culture was split into two portions again. Galactose was added to one portion (shown as Gal (ON)), and incubation was continued. Samples were taken at the indicated times (0–4 hours) and were analyzed by flow cytometry. B, Small aliquots of the same samples in A were taken and incubated in YPAD containing α factor for 60 minutes at 25°C. Next, the viability was measured as in Figure S1B. C, Whole cell extracts were prepared using the same samples in A and were analyzed by western blotting. Sld2 proteins were detected with anti-Sld2 antibody. The loading control shows the corresponding region of the Ponceau-S-stained membrane. *: non-specific background band. (TIF) [file pgen.1002136.s002.tif]

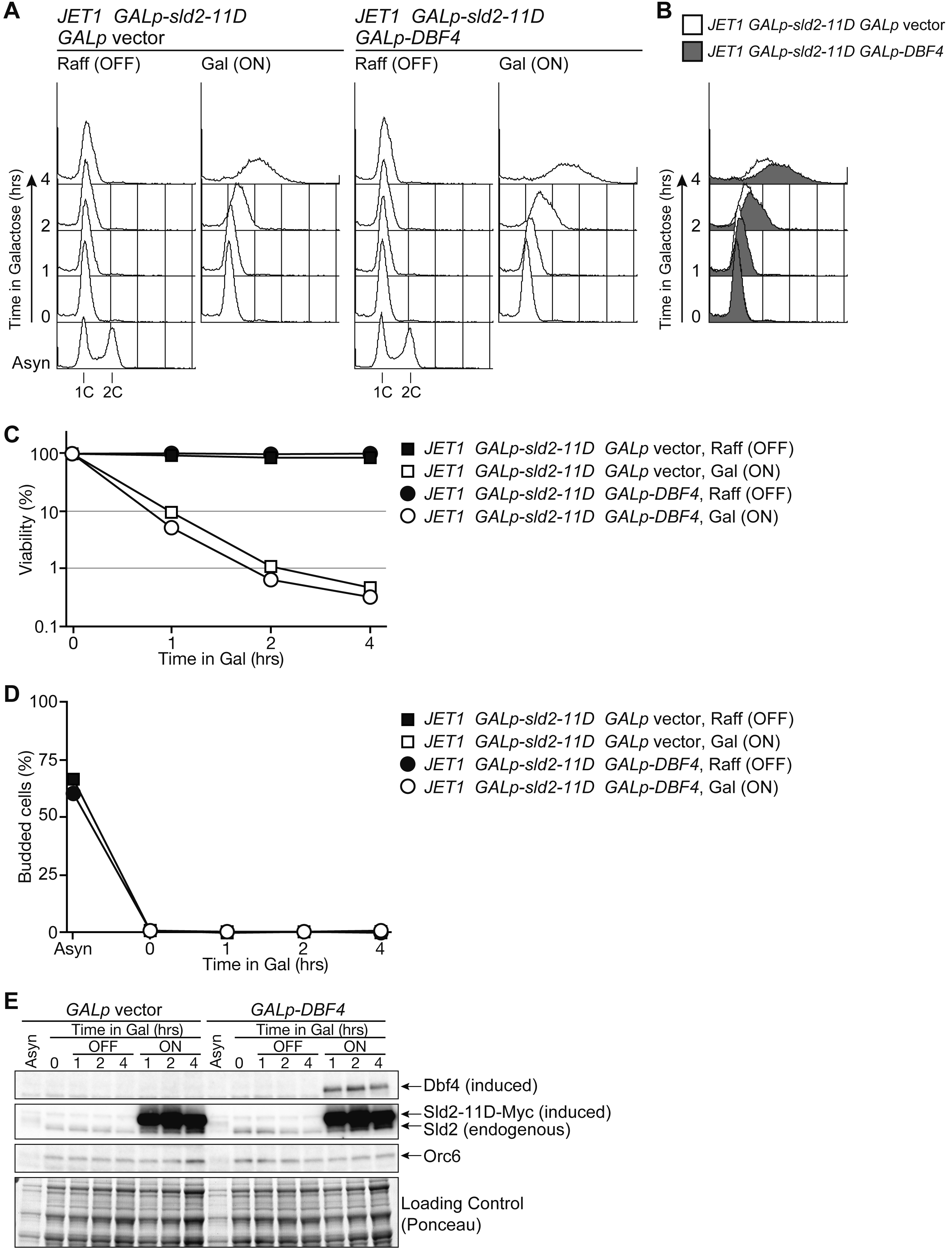

Supplement: Figure S3 — Dbf4 expression enhances the untimely initiation of DNA replication in G1. A, YST573 (JET1 GALp-sld2-11D GALp vector) and YST575 (JET1 GALp-sld2-11D GALp-DBF4) cells were grown in YPARaffinose medium at 25°C (Asyn) and arrested in G1 phase with alpha factor, and the culture was split into two portions. Galactose was added to one portion (Gal (ON)), and the incubation was continued. Samples were taken at the indicated times (0–4 hours) and analyzed by flow cytometry. B, An overlay of the flow cytometry profiles of A. C, Small aliquots of the same samples in A were taken, and the viability was measured as in Figure S1B. D, Proportion of cells with buds. C, Whole cell extracts were prepared from the same samples in A and were analyzed by western blotting. Sld2s, Orc6 and Myc-tagged Dbf4 proteins were detected with anti-Sld2, anti-Orc6 and anti-Myc antibodies, respectively. The loading control shows the corresponding region of the Ponceau-S-stained membrane. (TIF) [file pgen.1002136.s003.tif]

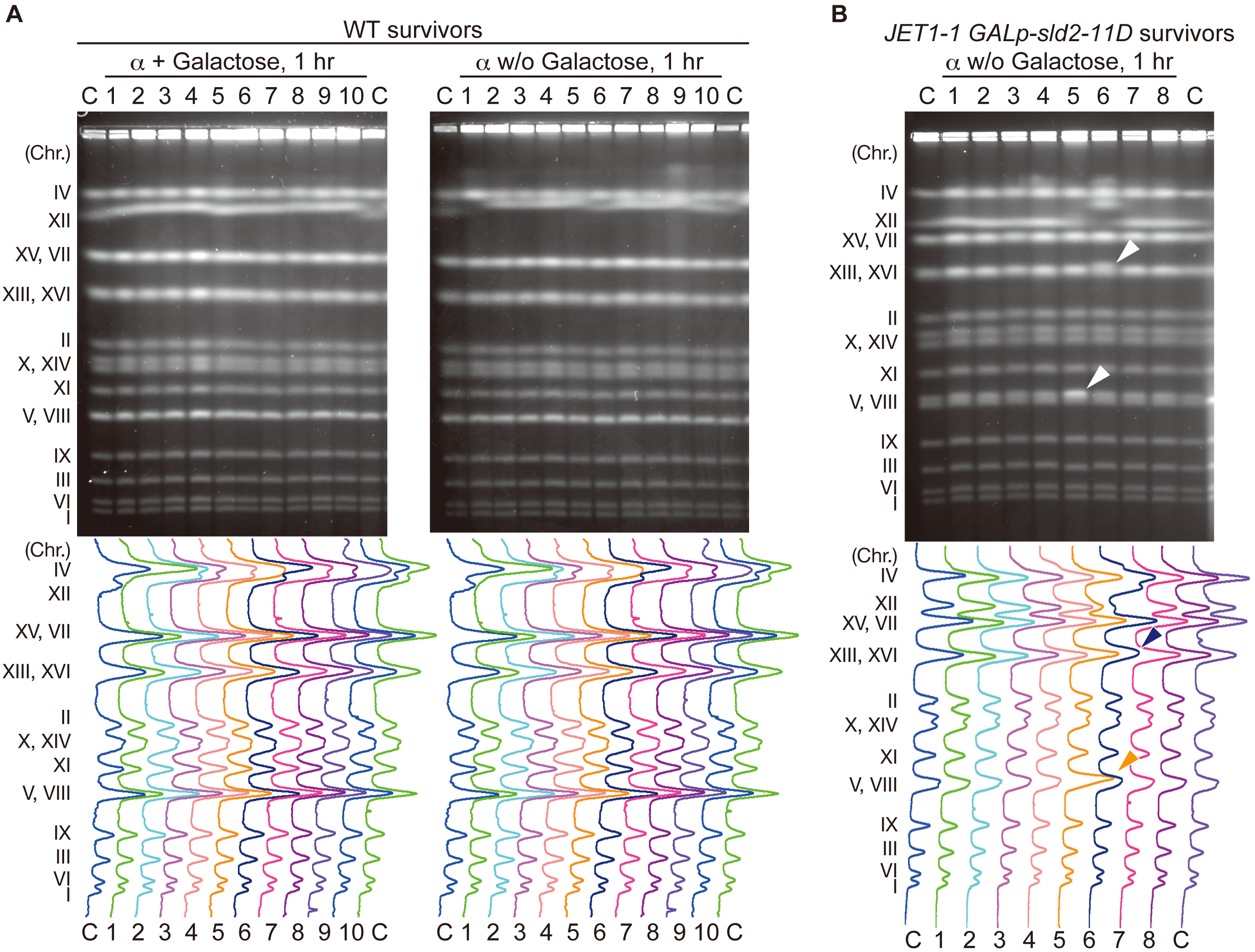

Supplement: Figure S4 — Control pulsed-field gel electrophoresis data for Figure 1. A, Chromosomal DNA from wild-type W303-1a Δbar1 colonies (WT survivors 1–10) that appeared on the YPAD plate was analyzed with pulsed-field gel electrophoresis. Before plating, cells were grown as in Figure S1A with (α+Galactose, 1 hr) or without (α w/o Galactose, 1 hr) galactose. The same cells were grown in YPAD and analyzed as a control (lane C). Quantified profiles for each lane are shown in bottom. B, The chromosomal DNA of the YST563 (JET1-1 GALp-sld2-11D) colonies (survivors 1–8) that appeared on YPAD plates after glucose incubation (Glc (OFF))) in Figure S1 was analyzed. Abnormal chromosome bands are indicated with arrowheads. (TIF) [file pgen.1002136.s004.tif]

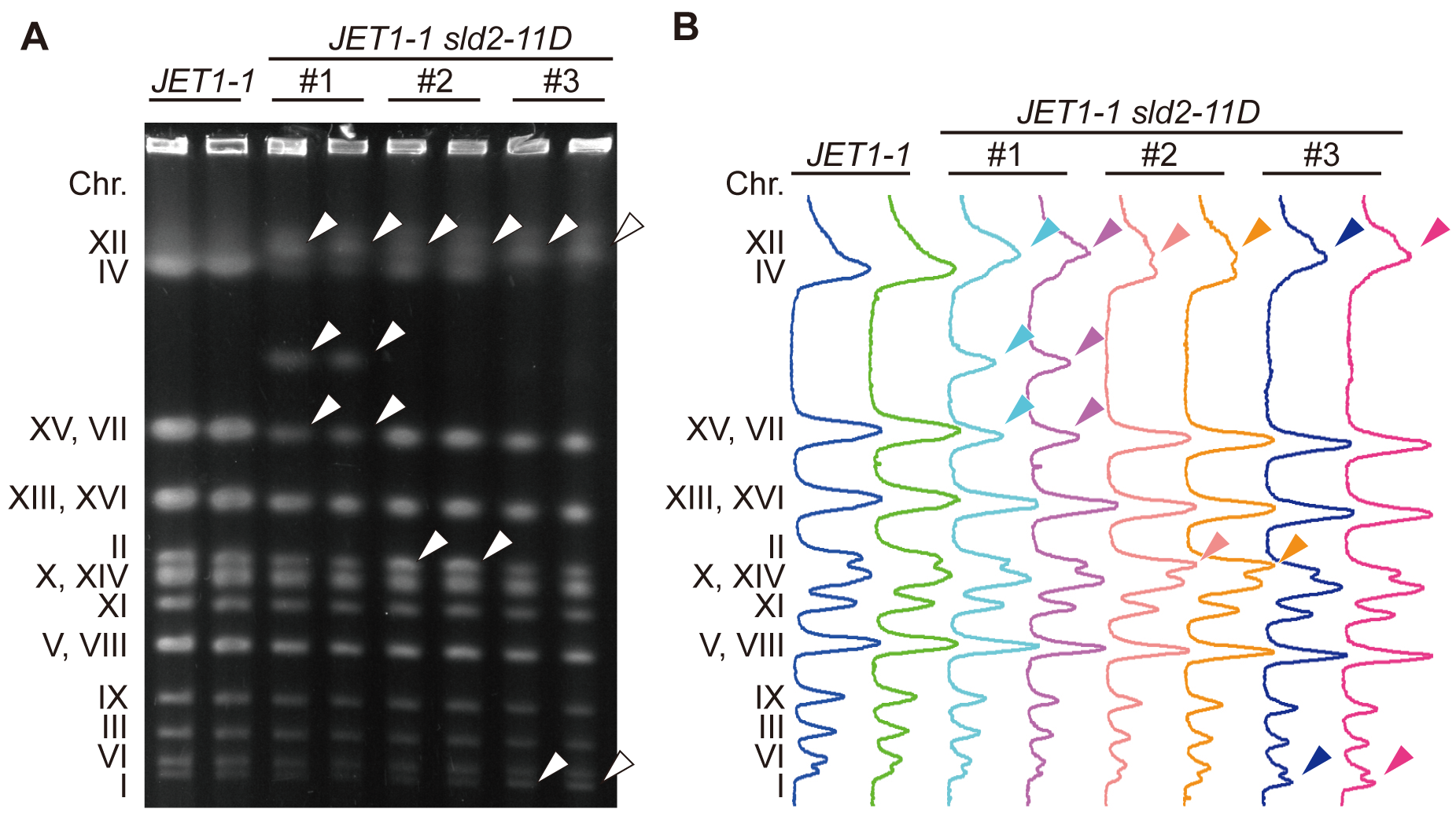

Supplement: Figure S5 — Chromosomes are unstable in CDC45JET1-1 sld2-11D cells. A, Chromosomal DNA from YST556 (JET1-1) YST819 (JET1-1 sld2-11D, #1), YST820 (JET1-1 sld2-11D, #2) and YST821 (JET1-1 sld2-11D, #3) was analyzed with pulsed-field gel electrophoresis. The same sample was loaded in duplicate for each strain. Abnormal chromosome bands are indicated with arrowheads. B, The profiles of the band intensities of A are shown. Abnormal chromosome bands are indicated with arrowheads. (TIF) [file pgen.1002136.s005.tif]
